# Potential Pathogenic and Opportunistic Oral Bacteria in Early Life: The Role of Maternal Factors in a Portuguese Population

**DOI:** 10.3390/pathogens12010080

**Published:** 2023-01-03

**Authors:** Mariana Fernandes, Maria João Azevedo, Carla Campos, Ana Filipa Ferreira, Álvaro Azevedo, Inês Falcão-Pires, Egija Zaura, Carla Ramalho, Joana Campos, Benedita Sampaio-Maia

**Affiliations:** 1 INEB—Instituto Nacional de Engenharia Biomédica, 4150-177 Porto, Portugal; 2 i3S—Instituto de Investigação e Inovação em Saúde, Universidade do Porto, 4200-135 Porto, Portugal; 3 Faculdade de Medicina Dentária, Universidade do Porto, 4200-393 Porto, Portugal; 4 Academic Center for Dentistry Amsterdam, University of Amsterdam and Vrije Universiteit Amsterdam, 1081 LA Amsterdam, The Netherlands; 5 Instituto Português de Oncologia do Porto Francisco Gentil, 4200-072 Porto, Portugal; 6 Escola Superior de Saúde, Instituto Politécnico do Porto, 4200-072 Porto, Portugal; 7 Cardiovascular R&D Centre—UnIC@RISE, Department of Surgery and Physiology, Faculty of Medicine, University of Porto, 4200-319 Porto, Portugal; 8 EPIUnit—Unidade de Investigação em Epidemiologia, Instituto de Saúde Pública, Universidade do Porto, 4050-600 Porto, Portugal; 9 Laboratório para a Investigação Integrativa e Translacional em Saúde Populacional (ITR), 4050-600 Porto, Portugal; 10 Obstetrics, Gynaecology and Pediatrics Department, Faculdade de Medicina, Universidade do Porto, 4200-319 Porto, Portugal; 11 Centro Hospitalar Universitário de São João, 4200-319 Porto, Portugal

**Keywords:** oral microbiome, maternal oral health, mother–child microbiome transmission, opportunistic/pathogenic bacteria, cardiovascular risk

## Abstract

In early life, maternal factors are of the utmost relevance for oral microbiome acquisition and maturation. Therefore, our study explored the impact of maternal factors, such as saliva and breastmilk colonization, cardiovascular risk factors (CRF), type of delivery, oral health, and caregiving habits on the prevalence of potential pathogenic and opportunistic oral bacteria in early life. A total of 26 healthy mothers, 23 mothers with CRF, and their 50 children were included and samples (child’s oral swabs, mother’s saliva, and breastmilk) were collected 4 to 12 weeks after delivery and inoculated in selective and differential media for detection of non-fastidious Gram-negative and Gram-positive bacteria to isolate potential pathogenic and opportunistic bacteria identified by MALDI-TOF MS (414 isolates). Within mother–child dyads, the same species were identified in 86% of the pairs and potential pathogenic microorganisms from the *Staphylococcaceae* and *Enterobacteriaceae* families were found to be statistically significantly concordant between mother–child samples, particularly in the healthy group. *Staphylococcus saprophyticus* and *Stenotrophomonas maltophilia* oral colonization in mother–child pairs were associated with the presence of CRF. Breastfeeding was related to the early life oral colonization of *Staphylococcus epidermidis* in children from healthy mothers and C-section was associated with higher diversity of pathogens, independent of cardiovascular status (*p* = 0.05). This study reveals the presence of potential oral opportunistic and pathogenic bacteria in early life and highlights the importance of maternal factors in its acquisition.

## 1. Introduction

The human microbiome is defined as a set of diverse microorganisms that inhabit specific niches of the human body such as the gut, the oral cavity, the skin, and the genitourinary tract and plays a key role in the balance between health and disease [1]. The host–microbiome dyad regulates paramount aspects of host physiology, metabolism, immunity, and neurologic function [2]. In contrast, dysbiosis represents an unbalance in the microbial community caused by changes in bacterial function and diversity or even a sudden fluctuation in the profusion of certain microbial populations [3]. These ecological shifts seem to contribute to the etiopathogenesis of several systemic conditions, such as obesity, arterial hypertension, or even gestational diabetes, all representing cardiovascular risk factors [4,5,6].

The oral cavity houses one of the most diverse microbiomes in the human body, harboring hundreds of species [7,8]. It encompasses a community of commensal, symbiotic, and pathogenic microorganisms including bacteria, archaea, protozoa, and viruses housed in distinct habitats, such as the teeth, gum, cheeks, and tongue [8]. Numerous studies have demonstrated over the years that oral dysbiosis plays a crucial role in the pathogenesis and development of the most common oral diseases such as dental caries, periodontal disease, and candidiasis, due to the presence of pathogenic and/or opportunistic microorganisms (e.g., *Streptococcus mutans, Staphylococcus aureus, Fusobacterium nucleatum, Porphyromonas gingivalis, Candida* sp.) [9,10,11,12]. In addition to the inherent risk of spreading to the various organs and tissues of the human body, these microorganisms can cause or aggravate systemic diseases, such as diabetes, obesity, or cardiovascular disease [8,13].

Regardless of the debate of whether bacteria are present in the placenta, it is currently recognized that the transmission of a wide variety of microorganisms to the newborn occurs at delivery [14,15,16]. Moreover, the type of delivery and breastfeeding have a high impact on the initial microbiota in infants as they directly influence the colonization in multiple human sites (gut, oral cavity, and skin) [17]. Furthermore, specifically regarding the colonization of the oral cavity, studies focused on the phenotypic and genotypic characteristics of oral microorganisms have demonstrated that the oral microbiota of the mother represents the most important route of transmission of the child’s oral microbiota in early life [18]. Thus, a mother presenting a dysbiotic microbiota may severely impact her child’s microbiota acquisition by prompting colonization by deviant microbiota such as opportunistic or pathogenic bacteria [19,20,21].

Nevertheless, knowledge of the impact of maternal factors such as the existence of maternal cardiovascular pathologies, oral health behaviors, and infant care habits on the infant’s microbiota acquisition and maturation is still scarce [8]. When comparing the information available on the impact of maternal factors on the children’s oral microbiota with the gut microbiota, a large contrast is noticeable. Therefore, due to the importance of the oral cavity in the surfacing of systemic diseases by pathogenic microorganisms and the lack of studies, we must fill this gap [22]. Thereby, this study aimed to detect and identify potential pathogenic and opportunistic oral bacteria in early life, verify their prevalence, and correlate bacterial profiles with maternal factors. Maternal factors include saliva and breastmilk colonization; cardiovascular risk factors (CRF) such as obesity, hypertension, and gestational diabetes; type of delivery; antibiotic use; oral health; and infant caregiving habits. To this extent, we pretend to verify a possible vertical transmission of microorganisms from mother to child and investigate what maternal factors may influence this process.

## 2. Materials and Methods

### 2.1. Participant Recruitment

This study was approved by the Centro Hospitalar Universitário de São João (CHUSJ) Ethics Committee for Health (Nº294/2018) and Unidade Local de Saúde de Matosinhos (ULSM) Ethics Committee for Health (86/CE/JAS). Recruitment of pregnant women was done at the Obstetrics Service of CHUSJ and ULSM. Exclusion criteria were age under 18 years old, pre-existing cardiomyopathy, kidney disease, chronic obstructive pulmonary disease, active systemic infection, and genetic syndromes. The study groups comprised: (1) healthy mothers (n = 26), without cardiovascular risk factors and their children, and (2) mothers with cardiovascular risk factors: arterial hypertension and/or obesity and/or gestational diabetes (n = 23) and their children. In the healthy mothers’ group, there was a pair of twins. For the purpose of analyzing the similarity between species found in mother and child, we consider the twins and their mother two mother–child pairs. All the participants were informed about the aim of the research and gave written consent prior to the study enrolment. The characterization of the mother–child pairs was performed through questionnaires that included questions related to maternal oral health, demographic data, clinical data, obstetric and perinatal outcomes, and habits that could promote vertical transmission from mother to child via saliva or breastmilk of microorganisms.

### 2.2. Sample and Data Collection

The mother–child pairs (n = 50) underwent saliva and breastmilk collection 4 to 12 weeks after delivery. A total of 5 milliliters of maternal unstimulated saliva samples were collected at least 1 h after a meal or mouth/toothbrushing by passively drooling into a sterile tube. The breastmilk was collected either with an electric pump or manually into a sterile collection tube, after discarding the first drops. The oral samples from babies were collected using 4 oral swabs rubbed on their tongues for 5 s (flocked swabs). All samples (n = 138) were collected between June 2019 and February 2021. The collected samples were immediately placed in ice and aliquoted in a 1:1 proportion with brain heart infusion (BHI) broth with 10% glycerol and preserved at −80 °C until further processing.

### 2.3. Sample Processing, Bacterial Isolation, and Identification

From each pair, 100 µL of the samples were inoculated in selective and differential culture media to isolate potential pathogenic and opportunistic microorganisms, namely, MacConkey agar (Liofilchem^®^, Roseto degli Abruzzi (TE), Italy) and Mannitol-Salt agar (Liofilchem^®^, Italy) used for the detection of non-fastidious Gram-negative and Gram-positive bacteria, respectively. In particular, MacConkey agar is used for Gram-negative enteric bacilli and Mannitol-Salt agar for staphylococci isolation. Then, the plates were incubated at 37 °C in aerobic conditions from 24 to 48 h. From each sample, all colonies presenting different morphologies, such as color, shape, and aspect, were selected (up to 6 isolates per plate) and further re-isolated in MacConkey agar and Mannitol-Salt agar.

All selected isolates were sub-cultured in BHI agar (Liofilchem^®^, Italy) plates and incubated at 37 °C to obtain fresh colonies (24 h). Each isolate was identified by Matrix-Assisted Laser Desorption/Ionization-Time of Flight (MALDI-TOF) mass spectrometry (MS) processed on the Bruker MALDI Biotyper^®^ system according to the manufacturer’s instructions (Bruker, MA, USA). Isolates were only considered to be identified if they obtained an identification score value equal to or higher than 2.0, corresponding to probable identification to species level.

### 2.4. Statistical Analysis

The statistical analysis was done with Statistical Package for the Social Sciences (IBM^®^ SPSS^®^ Statistics, SPSS Inc., Chicago, IL, USA, 28 version) for descriptive statistics and testing statistical hypotheses. All the results are represented as mean ± standard deviation (SD), median [interquartile range], or percentages (%). For comparison of frequencies, Pearson’s Chi-square test was used or, alternatively, Fisher’s exact test was applied when more than 20% of joint events displayed expected counts of less than 5 or even when any joint event presented expected counts of less than one. Concordance analysis was done by Kappa statistics to assess the similarity between matched groups. Continuous variables were described using mean ± SD. The normality assumption was assessed by Shapiro–Wilk test. The Friedman test and the Mann–Whitney U test were applied for comparison between matched groups and independent groups, respectively. For all analyses, statistical significance was assumed when *p*-values were less than 0.05. The Venn diagram was designed with PowerPoint (Microsoft Office Professional Plus 2019).

## 3. Results

The population studied included a total of 49 mothers (26 healthy and 23 with CRF) and 50 children, including one pair of twins. The CRF included hypertension (39.l%), obesity (47.8%), and/or gestational diabetes (26.0%), which occurred as isolated or concomitant. The demographic information of the mother, including age and educational level, mother’s antibiotic use, and oral health habits as well as the child’s antibiotic or probiotic therapies, type of delivery, child’s suctional habits, and habits potentiating the mother to microbial transmission to her child per study group are displayed in Table 1. No differences were observed between the study groups regarding clinical, nutritional, and caregiving habits (*p* > 0.05 for factors).

From the 50 mother–child pairs studied, 414 isolates were recovered and a total of 37 bacterial species were identified from mothers’ unstimulated saliva (n = 49 samples; n = 151 isolates) and breastmilk (n = 38 samples; n = 98 isolates) from the mothers and their children’s oral swabs (n = 50 samples; n = 165 isolates). A large diversity of potential pathogenic species, including “ESKAPE” pathogens (*Enterococcus faecium*, *Staphylococcus aureus*, *Klebsiella pneumoniae*, *Acinetobacter baumannii*, *Pseudomonas aeruginosa*, and *Enterobacter* species) [23], were identified in all samples. Table 2 shows the prevalence of each identified species per type of sample and study group. In comparison to the healthy group, the prevalence of *Staphylococcus saprophyticus* was found to be lower (or absent) in the oral cavity of the children of the CRF group and *Stenotrophomonas maltophilia* was also absent in the saliva of mothers from the CRF group, attaining statistical significance (*p* < 0.05).

**Figure 1:**
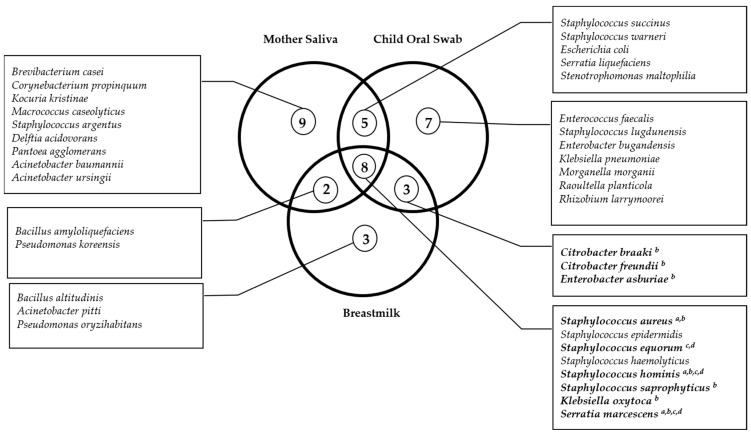
Venn diagram of the shared microorganisms between mother and child samples within mother–child pairs. Significant concordance evaluated by Kappa statistics (*p* < 0.05) between ^a^ mother saliva and child oral swabs in healthy mother–child pairs; ^b^ breastmilk and child oral swabs in healthy mother–child pairs; ^c^ mother saliva and child oral swabs in CRF mother–child pairs; ^d^ breastmilk and child oral swabs in CRF mother–child pairs.

We also explored the presence of similar species between mother–child pairs and verified that, in 86% (n = 43) of the pairs, the child presented the same bacterial species with the mother’s saliva and/or breastmilk. This analysis is also shown in Table 2 by species and by study group. From these, a total of 42.6% of the mother–child pairs presented the same species in all samples (child oral swab, mother saliva, and breastmilk), 38.3% of the pairs presented the same species between oral samples, and 19.1% presented the same species between oral swab and breastmilk. However, only regarding some species, we found statistically significant concordances between the child’s oral swab and the mother’s samples. This was the case of several microorganisms from the *Staphylococcaceae* and *Enterobacteriaceae* families where we found statistically significant concordances between the child oral swab and the breastmilk in the healthy group, but not in the CRF group, namely for *S. aureus* (Kappa statistics = 0.7; *p* < 0.001), *S. saprophyticus* (Kappa statistics = 0.4; *p* = 0.02), *Citrobacter braaki* (Kappa statistics = 1.0; *p* < 0.001), *Citrobacter freundii* (Kappa statistics = 1.0; *p* < 0.001), *Enterobacter asburiae* (Kappa statistics = 1.0; *p* < 0.001), and *Klebsiella oxytoca* (Kappa statistics = 1.0; *p* < 0.001). For *S. aureus*, concordance was found between the child’s oral swab and either the mother’s saliva or breastmilk, also only in healthy mother–child pairs (Kappa statistics = 0.8; *p* < 0.001). For two species, *Staphylococcus hominis* and *Serratia marcescens*, statistically significant concordance was found between the child oral swab and mother saliva or breastmilk either in the healthy or CRF group (*S. hominis* in breastmilk of the healthy group and child oral swab—Kappa statistics = 0.3; *p* = 0.005; *S. hominis* in mother saliva of the CRF group and child oral swab and Kappa statistics = 0.4; *p* = 0.03; *Serratia marcescens* in mother saliva of the healthy group and child oral swab—Kappa statistics = 1.0; *p* < 0.001, and *S. marcescens* in breastmilk of the CRF group and child oral swab Kappa statistics = 1.0; *p* < 0.001, respectively, for the CRF group). Only for *Staphylococcus equorum* was statistically significant concordance found between the three samples only in the CRF group (Kappa statistics = 1.0; *p* < 0.001, for both concordances) (Figure 1).

Moreover, we also investigated if maternal caregiving habits, such as breastfeeding, pacifier licking, and/or kissing on the lips, or other factors such as type of delivery, maternal antibiotic intake, maternal oral health habits, child’s antibiotics or probiotics therapies, and child’s suctional habits could impact the early life colonization of potential pathogenic and opportunistic bacteria. Breastfeeding appeared to promote the early life oral colonization of *Staphylococcus epidermidis* in children from healthy mothers (Fisher’s exact test; *p* = 0.05), while the other factors did not seem to impact the potential pathogenic and opportunistic bacteria of the child.

**Figure 2:**
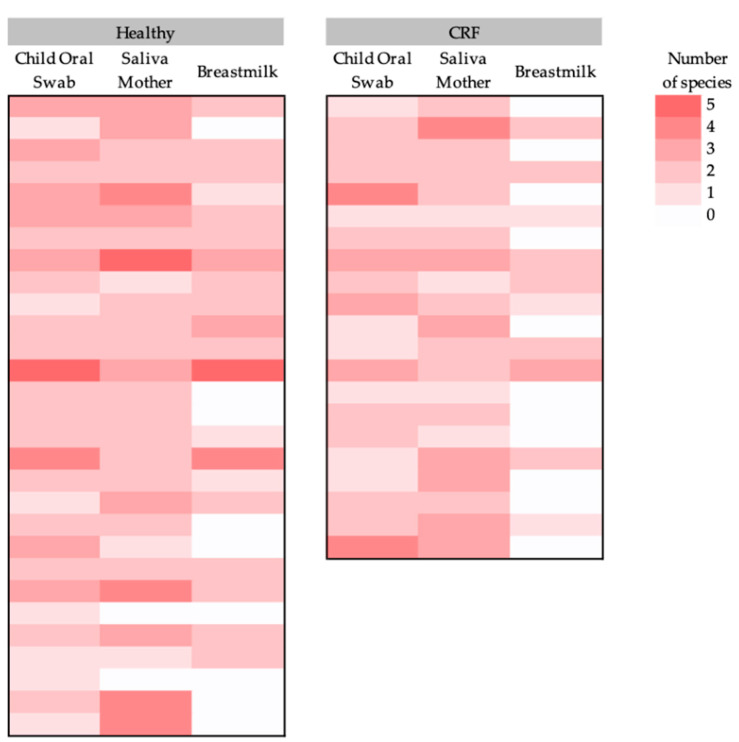
Heatmap of the (potential) pathogenic bacterial diversity (number of species per sample) per sample type (child’s oral swab, mother’s saliva, and breastmilk) for each study group (healthy women and women with CRF and their children).

The diversity of the potential pathogenic microorganisms in each sample varied between 0 and 5 species per sample (Figure 2). Shannon diversity was calculated and saliva samples from the mothers were the ones with the highest diversity, although this was not statistically significantly different from the other sources (child’s oral swabs, 0.2 ± 0.1 (mean ± standard deviation); mother’s saliva, 0.3 ± 0.1; breastmilk, 0.2 ± 0.2; Friedman test; Chi-square = 3.4; *p* = 0.2). Moreover, significant differences were found in the diversity of the child’s oral swab between the type of delivery, with children born by C-section having a higher diversity of pathogens (Mann–Whitney U test = 368.0; *p* = 0.05) (Supplementary Table S1). There were no significant differences in the diversity of potential pathogenic microorganisms found in the three sources types (child’s oral swab, maternal saliva, and breastmilk) between the cardiovascular status of the mother (in child oral swab: Mann–Whitney U test = 311.5; *p* = 0.9; in saliva mother: Mann–Whitney U test = 295.5; *p* = 0.8; and in breastmilk: Mann–Whitney U test = 232.5; *p* = 0.1) (Supplementary Table S1).

## 4. Discussion

The seeding and maturation of the child’s microbiome are essential processes to institute a healthy symbiosis between the host and microbiome. Moreover, the mother seems to play a crucial role in the transmission of microorganisms to the child. The process of oral colonization is influenced by manifold key factors including maternal comorbidities, mode of delivery, maternal antibiotic usage, gestational age at birth, feeding mode, and breastfeeding [17,24,25,26,27]. Furthermore, other factors such as maternal oral health behaviors and habits that predispose them to microbe transmission have been associated with early microbiome acquisition and succession in the infant [28]. Despite the vertical mother–child bacterial transmission arguably being the most paramount interplay in early life, the source and transmission routes by which the infants obtain these pioneering microorganisms remain largely underexplored [27].

In this study, there were differences regarding maternal cardiovascular health and the presence of certain pathogenic and opportunistic bacteria in the oral cavity of mothers, namely *S. maltophilia.* Likewise, *S. saprophyticus* was also significantly more prevalent in children from healthy mothers. *S. maltophilia* is increasingly recognized as an important cause of nosocomial and community-acquired infections, often associated with multidrug resistance [29,30]. A recent study suggests that the oral cavity may be a reservoir for this pathogen [31]. *S. saprophyticus* is a primary cause of community-acquired urinary tract infections mainly in sexually active women [32]. A study by Khadija et al. (2021) reported the presence of *S. saprophyticus* in the oral cavity of 11.98% of women postpartum, although the clinical relevance in the oral cavity is still unclear [33]. The role of this bacteria in the oral ecosystem of the child is yet to be clarified as well. Thereby, more studies are needed to better understand its oral colonization.

The majority of mother–child pairs (86.0%) presented similar bacterial species, and there were significant concordances between the same bacteria present in the child’s oral swab and the mother’s saliva and/or breastmilk, namely from the families *Enterobacteriaceae* and *Staphylococcaceae*. Curiously, some of these concordant microorganisms are considered “ESKAPE” pathogens and are already present in the oral cavity of children in early life. The acronym “ESKAPE” stands for *Enterococcus faecium, S. aureus, Klebsiella pneumoniae, Acinetobacter baumannii, Pseudomonas aeruginosa*, and *Enterobacter* species, a group of microorganisms representing a global threat to human health, due to a large number of infections in clinical and community settings, with limited therapy options [23,34]. This observation is in line with other works which report that the presence of potential pathogens in the oral cavity of children, such as *S. aureus* [35,36,37], *Pseudomonas aeruginosa* [38], and *Streptococcus mutans* [39,40], are possibly transmitted from their mother. Likewise, our study suggests that saliva and breastmilk are possible pathways of potential pathogen transmission between mothers and their respective children. Interestingly, the majority of the concordance in microbial presence between the mother and the child was found in the healthy group in comparison to the CRF group. Moreover, overall, healthy pairs showed a higher pool of potential pathogenic microorganisms, although this was not statistically significant. This fact may be related to two factors. First, our methodology focused on the search for non-fastidious potential pathogenic and opportunistic bacteria, and, second, a higher diversity of microorganisms is commonly associated with healthy states. In fact, cardiovascular diseases seem to be associated with a loss of microbial diversity of potential pathogenic taxa, which may be translated by the predominance of a certain genus of bacteria [41,42]. Maternal diversity may consequently impact the child’s health. Appropriate pre- and postnatal microbial stimulations are fundamental to training the neonatal immune system to correctly respond to the much larger inoculum transferred during delivery and breastfeeding [43]. Considering the importance of the child’s immune system stimulation with a diverse set of maternal microbes for its healthy maturation, if this microbial transfer is impaired because of maternal diseases, children may be predisposed to future immune diseases [44]. However, further studies are needed to understand the role of maternal health in the infant microbiome and immunity.

Regarding the mode of delivery, we found differences between the oral diversity of potential pathogenic bacteria in the children and the type of delivery, with children born by C-section harboring more potential pathogenic bacteria. Previous articles have demonstrated that infants born by C-section exhibit a reduced microbial diversity in saliva and have an increased susceptibility to immune and metabolic disorders such as obesity, diabetes, asthma, and allergies [45,46,47]. In agreement with our findings, a recent work on infant gut microbiome also reported that the presence of potential pathogenic and opportunistic species such as *Enterococcus*, *Enterobacter*, and *Klebsiella* spp. was higher in babies delivered by C-section [21]. Additionally, Mueller et al. (2021) observed that babies born by C-section had lower microbial diversity and a higher abundance of *Clostridium* species [48].

The feeding mode is an essential source of early microbial colonization and allows the maturation of the infant’s microbiome. In our study, there was a concordance in the presence of several microorganisms from the *Staphylococcaceae* and *Enterobacteriaceae* families in the oral cavity of the child and breastmilk, particularly in healthy mother–child pairs. Moreover, there was an association between breastfeeding and the colonization by *S. epidermidis* in children from healthy mothers. Breastfeeding introduces new microbial communities that stimulate growth, it functions as a microbiome modulator and is utmost to newborn development and health [49]. In fact, exclusively breastfed infants were reported to present an increased richness of *Streptococcus*, *Staphylococcus, Enterococcus*, and *Lactobacillus* [49]. Additionally, the pool of skin-associated microorganisms is reduced in formula-fed infants due to the lack of skin contact during breastfeeding [50,51]. Interestingly, a recent study observed an enrichment of *S. epidermidis* in caries-free children, suggesting a potential role for this species as a predictor of oral health [52]. Nevertheless, the influence of the feeding mode in the development and maturation of the gut microbiome is exceedingly more explored than in the oral microbiome, urging the need for more longitudinal studies [49,53].

Previous studies have shown that caregiving habits, such as licking the pacifier and kissing on the mouth, expose infants to parental oral microorganisms [54,55]. However, in our study, we did not observe associations between the presence of potential pathogenic bacteria and these behaviors. Still, given the importance of early oral colonization, larger studies are needed to better understand the real impact of specific maternal habits and behaviors on oral bacteria transmission to the infant and its influence on the child’s microbiome composition and diversity.

Regarding the limitations of this study, vertical transmission was not possible to be demonstrated in our study since the strain-level analysis of the recovered isolates was not performed. Despite MALDI-TOF MS being a well-known high-throughput and highly accurate method for microorganism identification, it is currently still unsuitable for the identification/differentiation of some highly related species [56]. Hence, further analysis with typing methods presenting higher discriminatory power should be performed, including confirming vertical transmission. It is also important to note that isolation of the oral streptococci community is quite challenging due to their nutritional/environmental requirements and, therefore, it was excluded from our analysis. Additionally, our work focused on bacteria identification, excluding pathogenic fungi. This should be addressed in the future as the oral cavity represents a well-known reservoir of potential pathogenic fungi [57]. Additionally, most participants self-reported their clinical information, smoking, drinking, oral hygiene, and caregiving habits, which may have introduced bias. The CRF group was also smaller in comparison to the healthy group, which may have also biased the results. For this reason, more longitudinal studies with larger cohorts of participants are required to reinforce the results from this work, where strain-level analysis (e.g., whole-genome sequencing) is performed. Moreover, to understand the potential pathogenicity of each of the isolated microorganisms, the detection of virulence factors should be performed, including the study of antimicrobial susceptibility, particularly against the most commonly prescribed antibiotics in dentistry.

Finally, our study suggests a relevant role for the mother as a source of potential pathogenic and opportunistic bacteria to the child in early life, and maternal factors, such as breastfeeding and delivery mode, seemingly impact the oral microbiota of the child. It is important to highlight that this study identified colonizers and potential pathogenic bacteria at the species level, bringing added value to the understanding of oral microbiome in early life, which nowadays is mostly characterized by genus or family levels in studies applying 16S rRNA methodology. Species identification is of utmost importance due to the different levels of potential pathogenicity of each microorganism, as well as their ecological relationships with the host. The evidence that the mother appears to be an important source of potential pathogenic microorganisms in early life, combined with the fact that the majority of these bacteria are involved in extra-oral diseases, reinforces the importance of maintaining good oral hygiene of mothers and caregivers as well as of children, particularly in immunocompromised infants. A recent meta-analysis by Ghaffari et al. (2018) [58] highlighted the effectiveness of both short- and long-term intervention programs on oral health-related attitudes, toothbrushing, and flossing among children during 3 months post-intervention. Therefore, oral health prevention programs must encompass the entire life span of an individual, starting with the mothers and caregivers and continuing from early life to old age.

## 5. Conclusions

In summary, in this study, we observed the presence of the same bacterial species in mother–child pairs, and hypothesized the occurrence of vertical transmission, via saliva and/or breastmilk, of potential opportunistic and pathogenic bacteria. Maternal factors appear to influence the acquisition of these potential opportunistic and pathogenic bacteria in early life. CRF affected not only the oral colonization of some bacteria in mother–child pairs but also the concordance of several bacteria from the *Staphylococcaceae* and *Enterobacteriaceae* families between mother–child samples, particularly in the healthy group. Additionally, breastfeeding appears to promote the early life oral colonization of *S. epidermidis* in children from healthy mothers and C-section appears to promote higher diversity of pathogens, independent of the cardiovascular status. Therefore, this study underlines the need to create awareness of the impact of maternal factors on the child’s oral microbiota acquisition.

## Figures and Tables

**Table 1:** Demographic and clinical information of the recruited healthy women (H) and women with cardiovascular risk factors (CRF) and their children.

	H	CRF
**Number of mothers, n (%)**	26 (53.0%)	23 (47.0%)
**Number of children, n (%)**	27 (54%)	23 (46%)
**Age (years) ^a^**	33.5 [27.8; 39.2]	35.0 [31.0; 39]
**Mother’s education level ^b^**		
Basic education	3.8%	4.3%
Secondary education	23.1%	43.5%
Bachelor degree	38.5%	43.5%
Master degree	26.9%	8.7%
Doctoral degree	7.7%	0.0%
**Mother’s oral health habits**		
Dental appointments (n/last year) ^a^	1.0 [0; 3.0]	2.0 [0; 4.0]
Frequency of toothbrushing (n/day) ^a^	2.0 [2.0; 2.0]	2.0 [1.0; 3.0]
Use of toothbrush complements * ^b^	84.6%	73.9%
**Mother’s antibiotic use**		
During pregnancy ^b^	7.7%	8.7%
Intrapartum ^b^	42.3%	52.4%
Postpartum ^c^	12.0%	4.3%
**Type of delivery ^b^**		
Vaginal	51.9%	66.7%
Cesarean	48.1%	33.3%
**Child’s therapy**		
Probiotic ^b^	40.7%	30.4%
Antibiotic ^c^	7.4%	4.3%
**Child’s feeding mode** **^b^**		
Breastfeeding	51.9%	45.5%
Formula	18.5%	18.2%
Breastfeeding and formula	29.6%	36.4%
**Child’s suctional habits ^c^**	88.5%	87.0%
Fingers/Hand	21.7%	20.0%
Pacifier	34.8%	30.0%
Fingers/Hand and pacifier	43.4%	50.0%
**Habits potentiating transmission**		
Pacifier licking by the mother ^c^	13.6%	11.8%
Child’s mouth kissing by the mother ^c^	11.5%	8.7%

Results are shown in prevalence (%) or median [interquartile range]. * The toothbrush complements include mouthwash, interdental brushes, and dental floss. **^a^** Mann–Whitney U test; **^b^** Chi-square test; ^**c**^ Fisher’s exact test.

**Table 2:** Prevalence of each species identified per sample type and study group (healthy (H) and with cardiovascular risk factors (CRF)) and prevalence of mother–child pairs presenting the same species (shared species) per study group.

Phylum	*Family*	Child Oral Swab		Mother Saliva		Breastmilk		Shared Species	
	*Species*	H	CRF	H	CRF	H	CRF	H	CRF
**Actinomycetota** **(Actinobacteria)**	***Brevibacteriaceae***								
	*Brevibacterium casei*	0.0%	0.0%	4.2%	0.0%	0.0%	0.0%	0.0%	0.0%
	***Corynebacteriaceae***								
	*Corynebacterium propinquum*	0.0%	0.0%	4.2%	0.0%	0.0%	0.0%	0.0%	0.0%
	***Micrococcaceae***								
	*Kocuria kristinae*	0.0%	0.0%	4.2%	0.0%	0.0%	0.0%	0.0%	0.0%
**Bacillota** **(Firmicutes)**	***Bacillaceae***								
	*Bacillus altitudinis*	0.0%	0.0%	0.0%	0.0%	5.6%	0.0%	0.0%	0.0%
	*Bacillus amyloliquefaciens*	0.0%	0.0%	0.0%	4.3%	0.0%	8.3%	0.0%	0.0%
	***Enterococcaceae***								
	*Enterococcus faecalis*	3.7%	0.0%	0.0%	0.0%	0.0%	0.0%	0.0%	0.0%
	***Staphylococcaceae***								
	*Macrococcus caseolyticus*	0.0%	0.0%	4.2%	0.0%	0.0%	0.0%	0.0%	0.0%
	*Staphylococcus aureus*	40.7%	21.7%	37.5%	60.9%	38.9%	16.7%	**33.3% ^a,b^**	10.5%
	*Staphylococcus argentus*	0.0%	0.0%	4.2%	0.0%	0.0%	0.0%	0.0%	0.0%
	*Staphylococcus epidermidis*	55.6%	65.2%	75.0%	87.0%	88.9%	83.3%	58.3%	78.9%
	*Staphylococcus equorum*	0.0%	4.3%	0.0%	4.3%	0.0%	8.3%	0.0%	**5.3% ^a,b^**
	***Staphylococcaceae***								
	*Staphylococcus haemolyticus*	3.7%	8.7%	4.2%	0.0%	0.0%	8.3%	0.0%	0.0%
	*Staphylococcus hominis*	48.1%	30.4%	0.0%	8.7%	33.3%	25.0%	**12.5% ^b^**	**15.8% ^a^**
	*Staphylococcus lugdunensis*	3.7%	21.7%	0.0%	0.0%	0.0%	0.0%	0.0%	0.0%
	*Staphylococcus saprophyticus*	**25.9%**	0.0% *	29.2%	4.3%	22.2%	16.7%	**12.5% ^a^**	0.0%
	*Staphylococcus succinus*	0.0%	8.7%	0.0%	4.3%	0.0%	0.0%	0.0%	0.0%
	*Staphylococcus warneri*	11.1%	0.0%	20.8%	13.0%	0.0%	0.0%	4.2%	0.0%
**Pseudomonadota** **(Proteobacteria)**	***Comamonadaceae***								
	*Delftia acidovorans*	0.0%	0.0%	4.2%	0.0%	0.0%	0.0%	0.0%	0.0%
	***Enterobacteriaceae***								
	*Citrobacter braaki*	3.7%	0.0%	0.0%	0.0%	5.6%	0.0%	**4.2% ^b^**	0.0%
	*Citrobacter freundii*	3.7%	0.0%	0.0%	0.0%	5.6%	0.0%	**4.2% ^b^**	0.0%
	*Enterobacter asburiae*	3.7%	4.3%	0.0%	0.0%	5.6%	0.0%	**4.2% ^b^**	0.0%
	*Enterobacter bugandensis*	0.0%	4.3%	0.0%	0.0%	0.0%	0.0%	0.0%	0.0%
	*Escherichia coli*	3.7%	4.3%	4.2%	4.3%	0.0%	0.0%	0.0%	0.0%
	*Klebsiella oxytoca*	3.7%	0.0%	4.2%	0.0%	5.6%	0.0%	**4.2% ^b^**	0.0%
	*Klebsiella pneumoniae*	0.0%	4.3%	0.0%	0.0%	0.0%	0.0%	0.0%	0.0%
	*Morganella morganii*	0.0%	4.3%	0.0%	0.0%	0.0%	0.0%	0.0%	0.0%
	*Pantoea agglomerans*	0.0%	0.0%	4.2%%	4.3%	0.0%	0.0%	0.0%	0.0%
	*Serratia liquefaciens*	0.0%	4.3%	0.0%	13.0%	0.0%	0.0%	0.0%	0.0%
	*Serratia marcescens*	3.7%	4.3%	4.2%	4.3%	0.0%	8.3%	**4.2% ^a^**	**5.3% ^b^**
	*Raoultella planticola*	0.0%	4.3%	0.0%	0.0%	0.0%	0.0%	0.0%	0.0%
	***Rhizobiaceae***								
	*Rhizobium larrymoorei*	0.0%	4.3%	0.0%	0.0%	0.0%	0.0%	0.0%	0.0%
	***Moraxellaceae***								
	*Acinetobacter pitti*	0.0%	0.0%	0.0%	0.0%	5.6%	0.0%	0.0%	0.0%
	*Acinetobacter baumannii*	0.0%	0.0%	0.0%	4.3%	0.0%	0.0%	0.0%	0.0%
	*Acinetobacter ursingii*	0.0%	0.0%	8.3%	4.3%	0.0%	0.0%	0.0%	0.0%
	***Pseudomonadaceae***								
	*Pseudomonas koreensis*	0.0%	0.0%	4.2%	0.0%	5.6%	0.0%	0.0%	0.0%
	*Pseudomonas oryzihabitans*	0.0%	0.0%	0.0%	0.0%	5.6%	0.0%	0.0%	0.0%
	***Xanthomonadaceae***								
	*Stenotrophomonas maltophilia*	3.7	0.0%	**20.8%**	0.0% *	0.0%	0.0%	**4.2%**	0.0%

* *p* < 0.05, significantly different in comparison to healthy women by Fisher’s exact test. **^a^** *p* < 0.05, significant concordance (Kappa statistics) between mother saliva and child oral swabs; **^b^** *p* < 0.05, significant concordance between breastmilk and child oral swabs.

## Data Availability

Not applicable.

## Supplementary Materials

The following supporting information can be downloaded at: https://www.mdpi.com/article/10.3390/pathogens12010080/s1, Table S1: Microbial diversity in each type of sample and potential impact of maternal factors.
