# Supplementary material for: Potential Pathogenic and Opportunistic Oral Bacteria in Early Life: The Role of Maternal Factors in a Portuguese Population
Source: Pathogens. 2023 Jan 3;12(1):80. doi: 10.3390/pathogens12010080 (PMC9867333; doi:10.3390/pathogens12010080)
Supplement: Supplementary file 1 [file pathogens-12-00080-s001.zip › pathogens-2086864-supplementary.pdf]

Supplementary Table S1: Microbial diversity in each type of sample and potential impact of maternal factors.

Type of samples	Maternal factor	Statistical test; p-value
Child's oral swab	Maternal CRF	Mann-Whitney U test=311.5; p=1.0
Saliva mother	Maternal CRF	Mann-Whitney U test=295.5; p=0.8
Breastmilk	Maternal CRF	Mann-Whitney U test=232.5; p=0.1
Oral swab diversity	Type of delivery	Mann-Whitney U test=368.0; p=0.05
